# Mosaic-Level Inference of the Impact of Land Cover Changes in Agricultural Landscapes on Biodiversity: A Case-Study with a Threatened Grassland Bird

**DOI:** 10.1371/journal.pone.0038876

**Published:** 2012-06-18

**Authors:** Francisco Moreira, João P. Silva, Beatriz Estanque, Jorge M. Palmeirim, Miguel Lecoq, Márcia Pinto, Domingos Leitão, Ivan Alonso, Rui Pedroso, Eduardo Santos, Teresa Catry, Patricia Silva, Inês Henriques, Ana Delgado

**Affiliations:** 1 Centre for Applied Ecology “Prof. Baeta Neves”, Institute of Agronomy, Technical University of Lisbon, Lisbon, Portugal; 2 Centre for Environmental Biology, Faculty of Sciences, University of Lisbon, Lisbon, Portugal; 3 Institute for Nature Conservation and Biodiversity, Lisbon, Portugal; 4 SPEA – Society for the Protection and Study of Birds, Lisbon, Portugal; Monash University, Australia

## Abstract

Changes in land use/land cover are a major driver of biodiversity change in the Mediterranean region. Understanding how animal populations respond to these landscape changes often requires using landscape mosaics as the unit of investigation, but few previous studies have measured both response and explanatory variables at the land mosaic level. Here, we used a “whole-landscape” approach to assess the influence of regional variation in the land cover composition of 81 farmland mosaics (mean area of 2900 ha) on the population density of a threatened bird, the little bustard (*Tetrax tetrax*), in southern Portugal. Results showed that ca. 50% of the regional variability in the density of little bustards could be explained by three variables summarising the land cover composition and diversity in the studied mosaics. Little bustard breeding males attained higher population density in land mosaics with a low land cover diversity, with less forests, and dominated by grasslands. Land mosaic composition gradients showed that agricultural intensification was not reflected in a loss of land cover diversity, as in many other regions of Europe. On the contrary, it led to the introduction of new land cover types in homogenous farmland, which increased land cover diversity but reduced overall landscape suitability for the species. Based on these results, the impact of recent land cover changes in Europe on the little bustard populations is evaluated.

## Introduction

Mediterranean ecosystems are amongst those ecosystem types predicted to undergo the greatest biodiversity changes in the long term [1]. The drivers for these changes include modifications in atmospheric carbon dioxide, climate, vegetation, and land use, but the latter is expected to play the main role [1]. In fact, the landscapes of the Mediterranean basin, particularly in Southern Europe, are changing at a fast pace (e.g., [2], [3]), with potential consequences for biodiversity that represent a major research topic (e.g., [4], [5]). In Mediterranean Europe, agricultural landscapes are particularly prone to change due to two major contrasting drivers: (i) abandonment of farming activities on marginal land, leading to loss of agricultural fields, shrub encroachment and afforestations of former agricultural land, and (ii) agricultural intensification in the most productive land, with consequences including the replacement of dry crops by irrigated crops, and loss of fallow land, pastures, and other non-crop habitats (e.g., [4], [6]–[8]).

Within Mediterranean Europe, vast regions of the Iberian Peninsula are covered by agricultural landscapes known as pseudosteppes, characterised by a mosaic of land covers including cereal crops, dry legumes, ploughed fields, and grasslands (pastures and fallows) [9], [10]. These land mosaics sustain populations of several bird species with unfavourable conservation status [6], [9]. One such species is the little bustard *Tetrax tetrax*, a medium-sized ground-nesting bird that has undergone a major decline in most of its Palaearctic range [11]. More than half of the world’s population now resides in the Iberian Peninsula [11], [12], where grasslands of different types (pastures, natural steppe and fallow fields) are its prime breeding habitat (e.g., [13]–[17]).

Little bustard populations, like those of other steppe bird species, are negatively impacted by both agricultural intensification and abandonment [10], [17], [18]. Both processes have impacts on land use and land cover patterns which result in changes in habitat availability and quality for the species. Thus, different farmland mosaic compositions are expected to drive regional variation in the population density of this species within its range. Studies in Spain and France showed a positive influence of land cover diversity on the occurrence of little bustard males (e.g., [13], [15], [18]). However, the Iberian regions where male densities are highest do not correspond to diverse landscapes, and are in fact dominated by vast expanses of grassland pastures or fallow land [12], [14], [19]. These latter findings are apparently in contradiction with the positive correlation of land cover diversity and male density found elsewhere.

Most of these previous studies were conducted at local scales, using properties of individual sites/patches or the landscape context surrounding each site as explanatory variables. However, to evaluate the implications for biodiversity of changes in land cover and land use it is necessary to understand the influence of the properties of whole land mosaics on the status of species, assemblages and ecological processes [20]–[22]. Agricultural landscapes are mosaics of different land covers and land uses which offer a range of habitats for plant and animal species [20]. These mosaics have properties that may influence animal populations, related to: (i) the total extent of a specific habitat of the target study species, (ii) the composition of the mosaic, a known driver of the species composition of faunal assemblages, and (iii) the spatial configuration of elements in the mosaic [20], which becomes more relevant as fewer patches of adequate habitat remain in the landscape [22].

In the present study, we used a “whole land mosaic” approach [20], [21] to explore the relationship between the regional variation in land mosaic composition and the density of little bustard. This type of evaluation, which can also be applied at the community level (e.g. biodiversity indices or species richness), requires landscape-level inference, which can be obtained only when both the response (population density) and explanatory variables characterise the landscape mosaic as a sample unit. In fact, landscape inference enables an assessment of the status of a species for the whole mosaic (not just a patch), and is more responsive to processes or patterns functioning at broad spatial scales [21]. Our aim here is to describe how regional variations in land mosaic composition influence little bustard densities, by characterizing land cover composition and little bustard density in 81 land mosaics in southern Portugal. More specifically, we hypothesise that little bustard male density should be higher in land mosaics dominated by the land cover providing adequate habitat for the species (grasslands) rather than to a higher land cover diversity.

## Materials and Methods

### Ethics Statement

No specific permits were required for the described field studies. Bird counts were carried out along public roads where no permission was required along private roads where, whenever possible, land owners were asked for permission. Little bustards are a protected species, but fieldwork was restricted to bird counts for which no specific permissions are required.

### Study Areas

**Figure 1:**
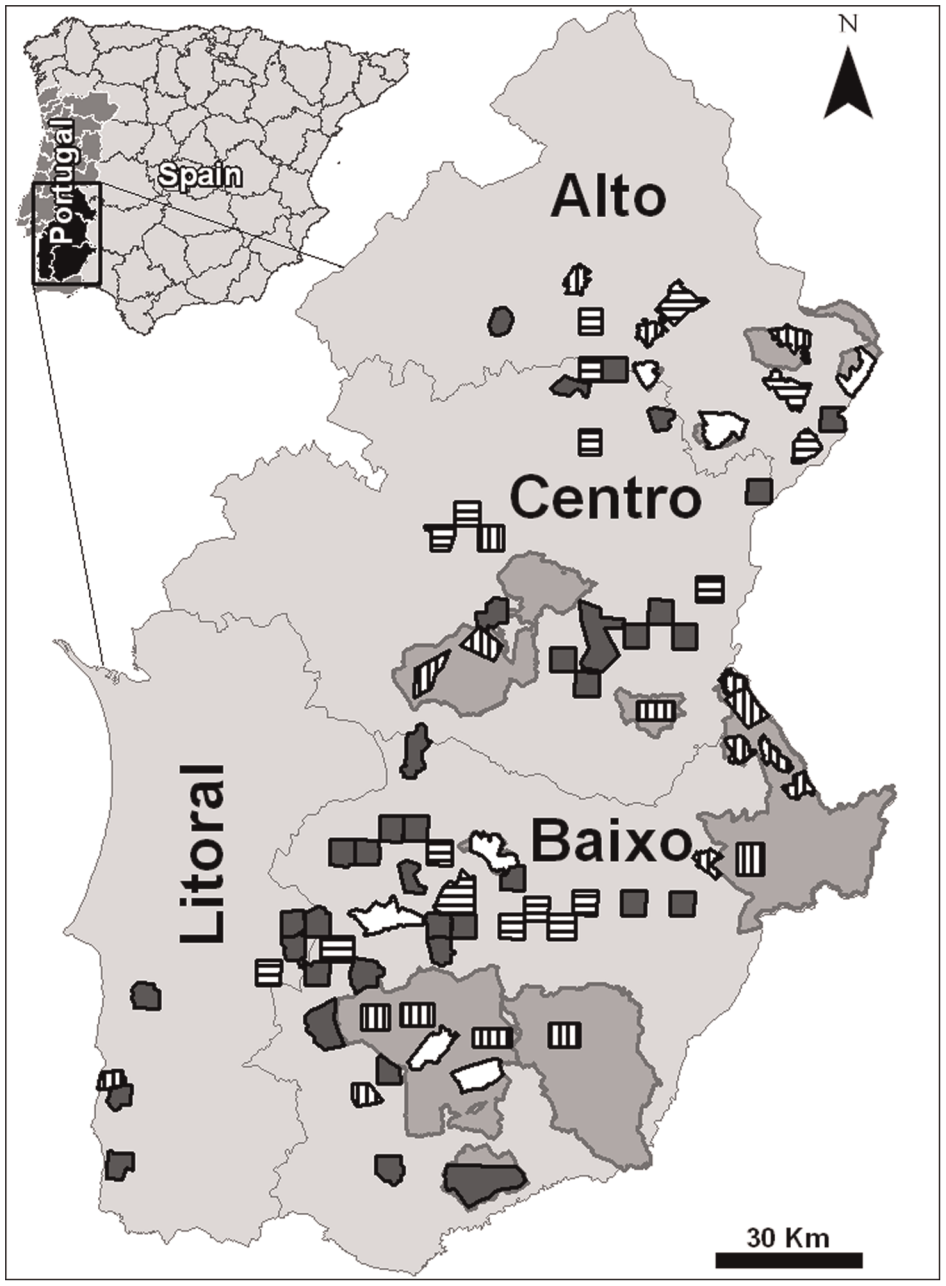
Location of the studied land mosaics for characterising little bustard densities in four regions of Alentejo (Alto, Centro, Baixo and Litoral), southern Portugal. Different stipple patterns correspond to different years of sampling: 2003 (white), 2004 (vertical pattern), 2005 (horizontal pattern) and 2006 (dark grey). Important Bird Areas (IBA) and Special Protection Areas (SPA) with importance for steppe birds are shown in light grey.

The Portuguese little bustard population is estimated at 10000–20000 individuals [11], mostly concentrated in the province of Alentejo, where we focused our sampling efforts. We defined 81 study sites (farmland mosaics) with areas ranging from 1657 to 9997 ha (mean = 2910 ha, median = 2502 ha, total area = 235740 ha) (Fig. 1), as follows.

Firstly, sites previously classified as Special Protection Areas (SPA) or Important Bird Areas (IBA) for steppe birds [23] (n = 14) were selected. These areas have a landscape mostly composed of open agricultural land, although a few also included forested and shrubland patches. Site limits corresponded to the total SPA/IBA area limits (or only the farmland portion, if the site also included forested areas) except if they were very large (over 10000 ha); in this case two to five mosaics of median size (1700 to 3600 ha) were defined in each SPA/IBA, as it would have been impossible to cover the whole area (due to logistic and time constraints).

Secondly, additionally to the IBA/SPAs, a set of 2500 ha land mosaics (n = 67) was defined by first overlaying a grid of 10×10 km squares over the Corine Land Cover map 1990 of the province of Alentejo (1∶100000) [24] and then selecting those squares with more than 40% of open agricultural and pastoral land area; land covers with potential little bustard habitat. In each of the selected 10×10 km units, we randomly selected two 5×5 km squares taking into account the level of sampling effort that could be made to cover the four sub-regions within Alentejo (Alto, Centro, Litoral and Baixo) (Fig. 1). Again, the total number of mosaics selected was dictated by the level of sampling effort that could be undertaken during a short time period (the male display period) every year.

The 81 sites were sampled for little bustards during 2003 (7 sites), 2004 (20 sites), 2005 (17 sites) and 2006 (37 sites). In each year, sampled sites were geographically stratified across the main sub-regions (Alto, Centro and Baixo), to avoid an association between year and sub-region (Fig. 1).

### Little Bustard Counts

In each of the 81 sites, little bustard density was evaluated using a network of point count locations covering the whole area. Firstly, we used 1∶25000 scale maps and field checks to identify the available road network that crossed each site. Following standard procedures to count little bustard males [12], [18], [26], points were then placed along the whole network of accessible non-paved roads crossing each sampling site, with the distance between survey points being set at a minimum of 600 m, to avoid double counts, and with the additional constraint that each point was at least 300 m from the site boundaries, villages and farmsteads, to minimise potential disturbance effects on little bustards. Because of differences in site area and road network density, the number of survey points per site ranged from 16 to 72 (mean = 29.1, total number of points = 2326). This corresponded to an average density of 1.05 points/km^2^ (median = 1, range = 0.48–1.54).

Each site was surveyed one to three times (mean = 1.4, median = 1) during April and May, which corresponds to the time of the breeding season when males are most active and conspicuous [13]. The number of counts was dictated by logistic constraints. Roads were travelled by car, and the total number of males detected within 250 m of each survey point (an area of 19.6 ha) during 5 minutes was recorded. This radius was selected because it is the distance at which any calling male is most likely to be detected, and this survey method has been widely used in other studies [16], [25], [26]. GPS point coordinates and a map (or aerial photographs) overlaid with the boundary of the search circles facilitated the assessment of the survey area and the bird counts. Visual confirmation of males detected by their calls was made to ensure that they were within the 250 m radius of the survey point. Birds that flew from the sampled area as the observer approached the survey point were also counted, after checking their landing location to avoid double counts. All surveys were carried out within the first three hours after dawn or three hours before dusk, coinciding with the known peaks in male activity [13], [27]. When more than one count was carried out per site, there was at least a one-week interval between successive counts. The sequence of road itineraries was changed from count to count to keep points from being sampled systematically at the same time of the day.

### Land Cover Composition

The landscape composition in each site was determined by overlaying the CORINE Land Cover map for 2006, derived from SPOT 4 satellite imagery [28] with site boundaries in a Geographic Information System (GIS). For the purposes of this study, the level-3 land cover nomenclature was simplified into seven classes (Table S1). Other land cover categories were much scarcer in the study areas and did not include potential habitats for little bustards (they mostly belonged to categories “artificial surfaces” and “wetlands”), and were discarded from the analyses.

One of the drawbacks of using the CORINE classification system is that it does not allow the distinction of the different uses within dry crops, the most common land cover type in the sampled sites (mean cover = 58.7%, median = 63.2%, range = 0.5–98.9%, n = 81). This information is highly relevant, as this broad category includes both uses that are highly suitable for breeding little bustards (such as pastures and fallow land) and less suitable ones (e.g. ploughed fields, cereal crops, and sunflower fields) [13], [29], [30]. To overcome this problem, the dry crop information from CORINE mapping was complemented with information collected during the field surveys. For this purpose, in each sampled point the land cover composition in the surrounding buffer was visually estimated to the nearest 12.5% by dividing the 250-m radius circle in 8 “slice” sections and recording the dominant land cover (covering the largest proportion of the area) in each section, for the following categories: (1) *grassland*s (fallow fields, permanent grasslands, and set-aside fields), (2) *ploughed fields*, (3) *cereal and hay fields*, (4) *dry legumes* (including chick pea and alfalfa). In most cases, the dominant cover was easily identified due to the large size of the fields. In sites counted more than once, the cover could change from count to count due to agricultural activities (e.g. ploughing of fields), thus availability was averaged. The relative proportion of these four land uses was estimated for each site and these estimates were used to replace the estimated total area covered by dry crops (derived from CORINE) by its four components (see Table 1).

**Table 1:** Explanatory variables and descriptive statistics (mean and range) across the 81 sampled sites.

Variable (short name)	Description	Mean (range)
Grasslands (Grass)	Proportion of grasslands, derived from CORINE corrected by field data	0.342 (0.001–0.776)
Cereal (Cereal)	Proportion of cereal, derived from CORINE corrected by field data	0.208 (0–0.620)
Irrigated crops (Irrigcrops)	Proportion of irrigated annual crops, derived from CORINE	0.123 (0–0.955)
Agro-forestry (Agrof)	Proportion of agro-forestry systems, derived from CORINE	0.104 (0–0.452)
Permanent crops (Permcrops)	Proportion of permanent crops, derived from CORINE	0.090 (0–0.400)
Shrublands (Shrub)	Proportion of shrublands, derived from CORINE	0.039 (0–0.266)
Ploughed (Plough)	Proportion of ploughed fields, derived from CORINE corrected by field data	0.031 (0–0.142)
Forests (For)	Proportion of forests, derived from CORINE	0.027 (0–0.214)
Mixed systems (Mixed)	Proportion of mixed systems, derived from CORINE	0.016 (0–0.191)
Dry legumes (Dryleg)	Proportion of dry legume crops, derived from CORINE corrected by field data	0.005 (0–0.074)
Richness (Rich)	Number of land cover types	6.8 (4–10)
Equitability (Equit)	Equitability of land cover types	0.73 (0.15–0.93)

Land cover variables are ordered by decreasing mean.

### Data Analyses

Estimates of male density obtained for each point were averaged to yield a mosaic-level density, expressed as males/km^2^, for each of the 81 sites. For sites sampled more than once, the mean male density was estimated for each sample point before estimating the mosaic-level average density. The existence of spatial dependence in the pattern of regional variation in male density was tested through a spatial correlogram based on the Moran’s I autocorrelation coefficient [31], using function *correlog* of the *ncf* package [32] of the R language [33]. Its significance was tested using 1000 permutations and the progressive Bonferroni correction [34].

For each site, in addition to the 10 variables expressing land cover, we calculated two land cover diversity estimates: (i) land cover richness, expressed as the number of different land cover types in the site, and (ii) land cover equitability, estimated as the Shannon diversity index divided by its maximum possible value (natural log of the number of land cover types). Equitability expresses the relative proportion of all existing land covers in a given site, and varies between close to 0 (when one land cover is vastly dominant) and 1 (when all land covers occur in similar proportions).These two variables were estimated using the *vegan* package [35] for R. As some of the 12 variables used for describing land cover composition were intrinsically interdependent (multicollinearity), we used principal components analysis (PCA) based on a correlation matrix to describe the main land cover gradients [34]. The angular transformation was applied to variables expressing proportions prior to PCA. We retained only Principal Components (PCs) with an eigenvalue larger than 1, as factors with variances smaller than unity are no better than a single variable. These new PC variables (expressing site coordinates in the selected components) have the advantages of being uncorrelated with each other and of summarizing most of the information contained in the original variables. To obtain simpler and more interpretable components, the factors were rotated using the varimax criteria, thus minimizing the number of variables with high loadings on a given factor [34]. The site coordinates along the PCs were mapped in the GIS, to visualise the spatial patterns of land cover composition across the region.

To model the influence of landscape composition (PC) variables on little bustard density, we used mixed effects models [36]. Function *lme* of the *nlme* package [37] was used to fit the mixed models in R, with year as a random effect. We followed Zuur et al. [38] (chapter 5) and started with a model where the fixed component contained all explanatory variables and different variance structures for the random part were sought, owing to heterogeneity in residuals. Once the optimal random structure was found, model building then concentrated on finding the optimal fixed structure (using Maximum Likelihood estimation), by backward selection of variables, based on likelihood ratio tests. The final model was re-run using restricted maximum likelihood estimation (REML). Model assumptions and model fit of the final model were assessed using the proportion of variance explained (r^2^), histograms and qqplots of residuals, and plots of residuals versus fitted values and explanatory variables. The existence of spatial dependence in residuals was tested through a spatial correlogram based on the Moran’s I.

## Results

### Regional Variation in Little Bustard Densities

**Figure 2:**
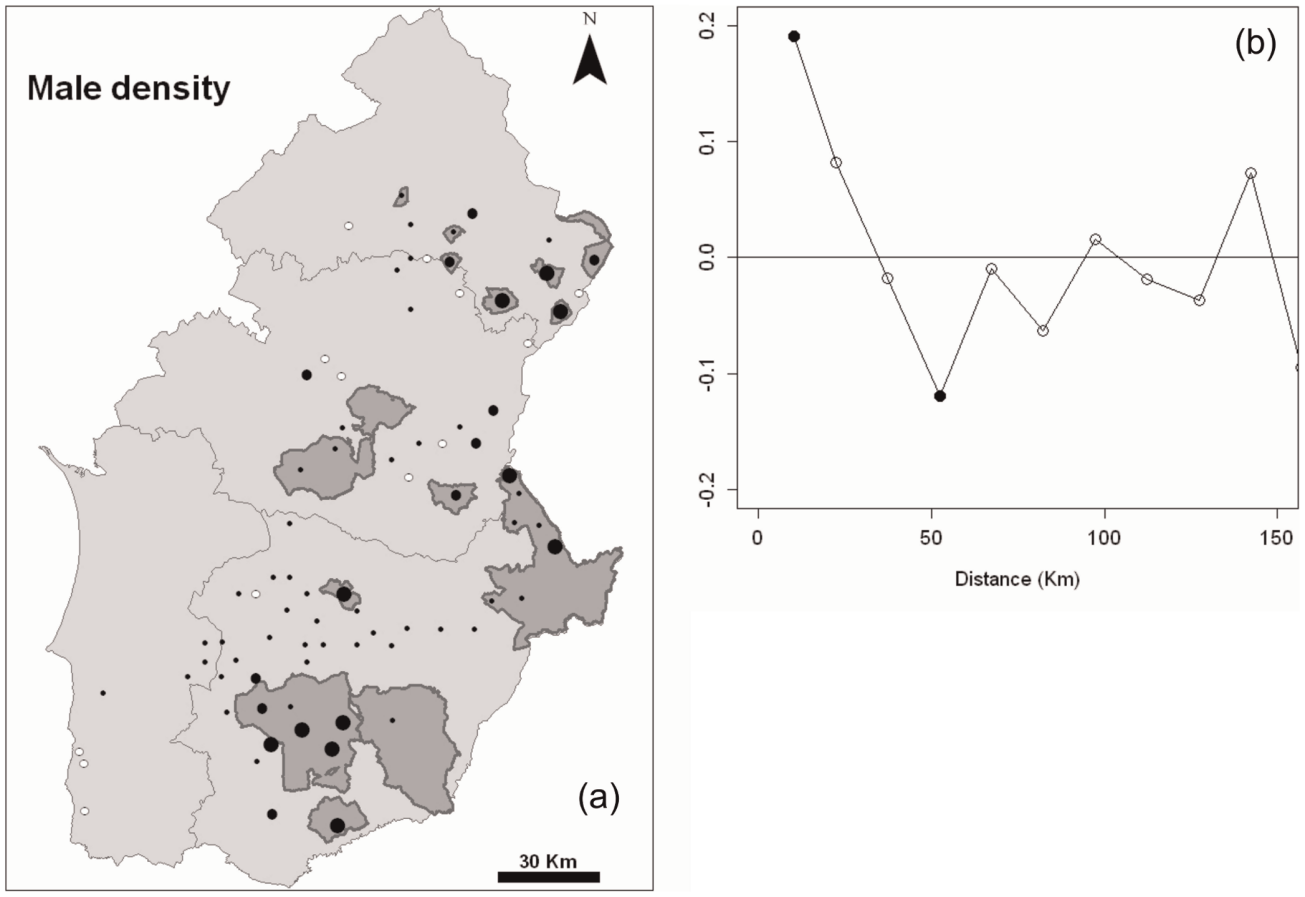
Spatial autocorrelation in little bustard density patterns. (a) Little bustard male density across the studied land mosaics in Southern Portugal. Important Bird Areas (IBA) and Special Protection Areas (SPA) with importance for steppe birds are shown in dark grey. Codes for male densities: small white dots (no males recorded), small black dots (0.01–2.99 males/km^2^), medium-sized black dots (3.00–4.99 males/km^2^), and large black dots (5.00–9.73 males/km^2^). (b) Spatial correlogram of little bustard male densities. Dark symbols represent correlation statistics significant (p<0.05) after progressive Bonferroni correction.

Little bustards were present in 68 of the 81 sampled land mosaics (Fig. 2A), and male regional density ranged from 0 to 9.73 males/km^2^ (mean = 2.25±0.258, n = 81). Sites with higher density (>5 males/km^2^) were concentrated mainly in Baixo and Alto Alentejo (see Fig. 1). The species was scarce or absent in Litoral Alentejo and in parts of Central Alentejo. A significant, although weak, positive spatial autocorrelation in bustard densities existed in nearby sites until a lag distance of ca. 10 km, and it declined progressively until a significant negative value was registered at lag distance of ca. 50 km (Fig. 2B).

### Regional Variation in Land Cover Composition

The most common land cover type in the studied sites was grassland, which occupied in average ca. 34% of the total area (Table 1). Cereal, irrigated crops and agro-forestry systems all had a mean coverage higher than 10%. The mean number of land cover types per site was 6.8, and mean land cover equitability was 0.73 (Table 1).

**Figure 3:**
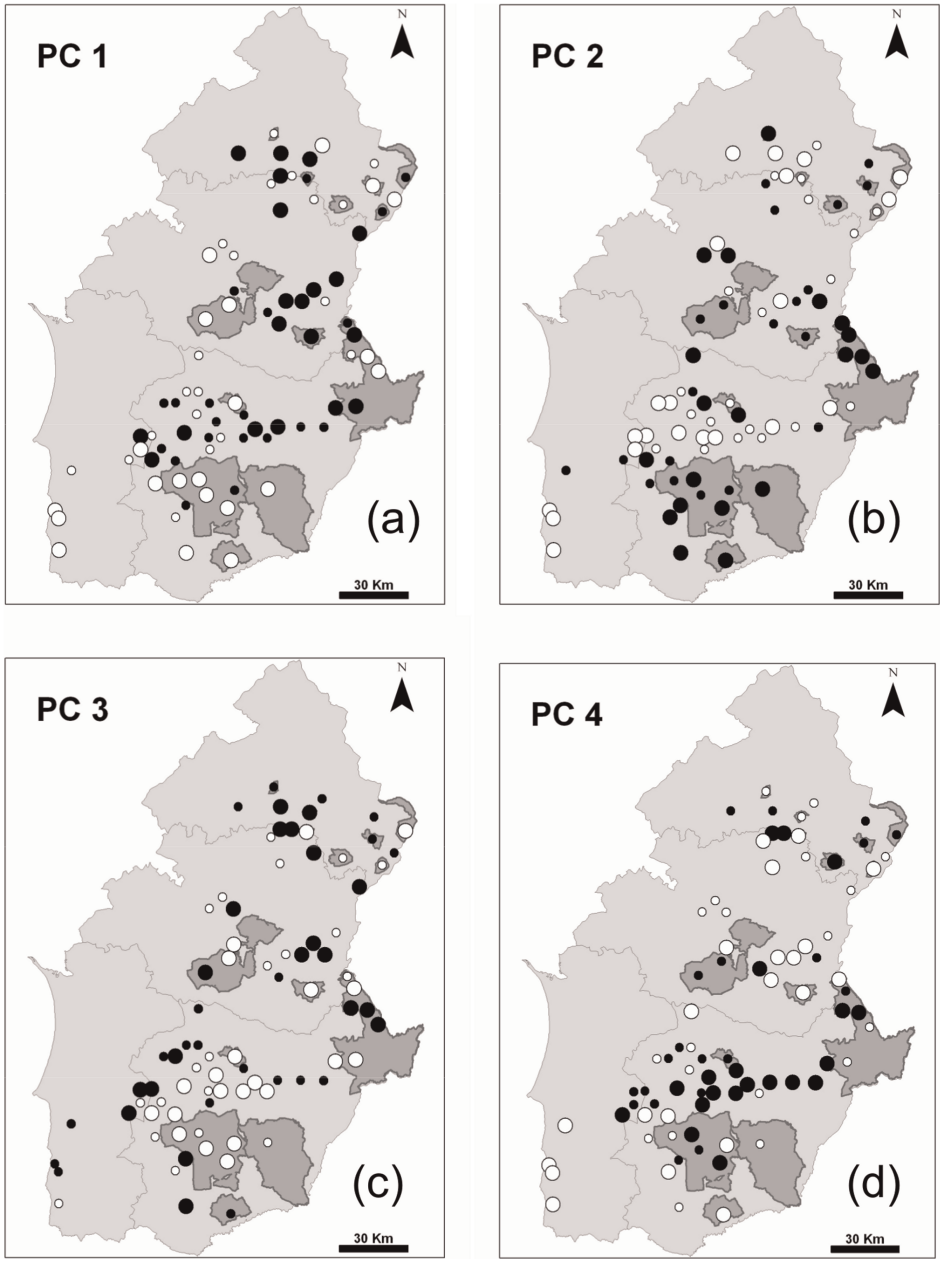
Site coordinates along the four first axes of a Principal Components Analysis to summarise land cover information in the 81 study sites. For each axis, each symbol denotes the four quartiles of site coordinates: large white dots (first quartile), small white dots (second quartile), small black dots (third quartile) and large black dots (forth quartile). (a) PC 1; (b) PC 2, (c) PC 3, (d) PC 4.

The 12 original variables were summarised into four Principal Components (Table 2) with an eigenvalue larger than 1, and these accounted for 63.7% of total data variance. The first PC (PC1) represented a gradient of sites ranging from low to high land cover diversity (expressed as richness and equitability), where this increase was also associated to a higher cover by permanent crops and mixed systems. The spatial distribution of the PC 1 scores (Fig. 3A) showed a concentration of sites with low land cover diversity on southern sites and a few clusters in Centro and Baixo Alentejo. PC 2 represented a gradient ranging from sites with a high proportion of irrigated crops to sites with more grasslands. The spatial distribution of the PC 2 scores (Fig. 3B) showed that sites with more irrigated crops occurred along the coast of Litoral Alentejo, the western and central part of Baixo Alentejo and also in specific sites in Centro and Alto Alentejo. The third PC represented a gradient ranging from sites with higher proportions of cereal and ploughed fields to sites with more forests and agro-forestry systems. The spatial distribution of the PC 3 scores (Fig. 3C) showed a large cluster of sites with more cereal and ploughed fields in Baixo Alentejo. The fourth PC was mostly a gradient of decreasing proportion of shrublands and increasing proportion of legume fields. Shrublands were more common in Litoral Alentejo, southern Baixo Alentejo and Centro Alentejo (Fig. 3D).

**Table 2:** Principal component loadings, eigenvalues and explained variance (% var.) for varimax rotated PC axes 1 to 4 describing patterns in land cover composition across the 81 study sites.

variable	PC 1	PC 2	PC 3	PC 4
Rich	**0.82**	-0.05	-0.09	-0.13
Permcrops	**0.71**	-0.26	0.05	0.31
Equit	**0.65**	0.40	0.13	0.23
Mixed	**0.51**	0.24	-0.06	-0.50
Irrigcrops	-0.15	**-0.89**	0.06	-0.04
Grass	-0.39	**0.69**	-0.07	-0.16
Plough	0.16	0.02	**-0.72**	0.04
Agrof	0.29	0.45	**0.68**	0.16
Cereal	0.24	0.18	**-0.68**	0.47
For	0.39	-0.21	**0.65**	-0.15
Shrub	-0.14	0.25	0.13	**-0.63**
Dryleg	-0.00	0.21	-0.06	**0.53**
				
Eigenvalue	2.371	1.949	1.929	1.392
% var.	19.7	16.2	16.0	11.6

Variables with correlation coefficients higher than 0.50 are highlighted in bold.

### Land Cover Predictors of Little Bustard Density: Model Building

The initial model (AIC = 335.1, Table S2), including the random factor (year) and the 4 PC variables, showed heterogeneity in the residual patterns, mainly because of an increased residual spread along with PC 2 as well as different residual spread per year. Thus, residual heterogeneity was allowed by exploring different variance structures [38]. The comparison of several alternatives (Table S2) showed that the model including a combination of variance structures allowing a different spread per year and an exponential increase with PC 2 had the lowest AIC (324.0) and represented a significant improvement compared to the initial model (Likelihood ratio test = 19.1, p<0.001).

**Figure 4:**
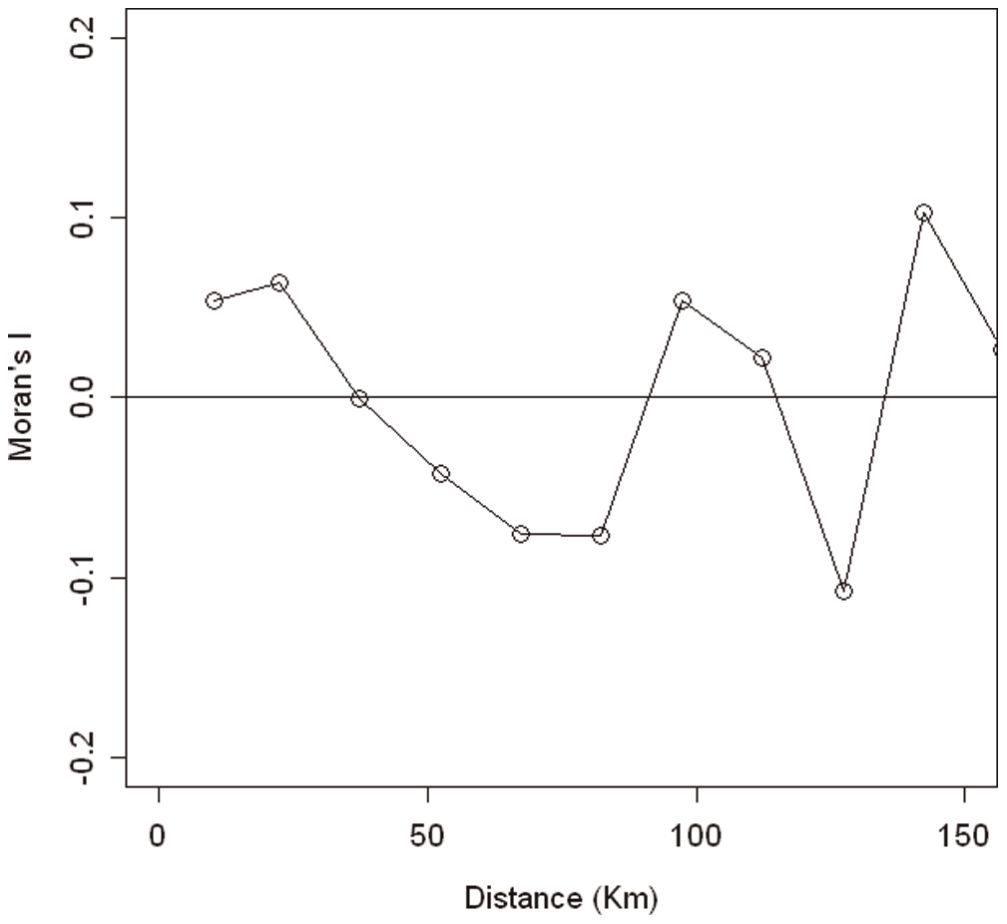
Spatial correlogram of the normalized residuals of the mixed effects model of the relationships between little bustard male densities and land cover variables.

There was no significant spatial autocorrelation in both initial and varComb model residuals at any lag distance, showing that the existing spatial correlation in male densities was induced by exogenous processes [31], [34], namely the similarities in land cover composition in nearby sites. Thus, the explanatory variables in the model effectively accounted for the spatial dependence. Model building then continued with the fixed part, where the backward selection of the variables resulted in a model with the first three PCs. In this final model, the random component results showed that the random intercept had a variance of 0.50 and the correlation between sites sampled in a given year was quite low (intraclass correlation was 0.08). The estimates for the separate standard deviations per stratum (year) showed that residual variability was the highest in 2005 and the lowest in 2006. Finally, residual spread increased also as a function of e^(0^.^82* PC 2)^. In the fixed component, the results (Table 3) showed that the more important variable explaining bustard density was PC 2, with densities positively correlated with this variable, meaning that the species was more abundant in land mosaics dominated by grasslands and with lower proportion of irrigated crops. Both PC 1 and PC 3 had negative coefficients, showing that higher densities were attained in mosaics with lower land cover diversity (and less permanent crops and mixed systems) and a lower proportion of forests and agro-forestry systems (and more cereal and ploughed fields). This model explained 48% of the regional variability in little bustard density, and there was no significant spatial autocorrelation in the residuals (Fig. 4).

**Table 3:** Coefficients of explanatory variables (land cover PCs) (± standard errors) in the fixed part of the linear mixed model, and their significance.

Variable	Coefficient	P-value
PC 1	-0.48±0.142	0.0011
PC 2	0.73±0.111	<0.001
PC 3	-0.46±0.161	0.0054

Model AIC = 308.7 and r^2^ = 48.1%.

## Discussion

In the present study, we used a “whole land mosaic” approach [20] to explore the relationship between the regional variation in land cover composition and the population density of a threatened bird in 81 land mosaics spread across southern Portugal. This large scale approach provides the best evaluation of biodiversity or population responses to changing land cover composition, the main driver of biodiversity changes in Mediterranean landscapes, and is recommended for conservation strategies for landscape mosaics [22]. However it is seldom used, at least in agricultural landscapes [20]. One assumption of this approach is that the mosaic-scale density of little bustards is a reliable indicator of landscape suitability, which may not be the case for all species [39].

### Regional Variation in Male Densities

The widespread occurrence of the little bustard and the population densities measured in Alentejo suggest that, within an Iberian context, the region as a whole is suitable for the species. In fact, the regional density of ca. 2 males/km^2^ estimated in the current study is similar to that observed in many areas in Spain (e.g., [21], [25], [29]), and is well above the densities observed in Western France [40]. Exceptional regional mean densities of over 5 males/km^2^, rare in other regions of the Iberian Peninsula, occurred at 12 land mosaics, of which four were in the Castro Verde region, where a population of 3,400 to 5,000 males was estimated [41]. There was spatial autocorrelation in measured densities, with nearby sites (until ca. 10 km away) tending to share a high or low density of little bustard males. This spatial dependence could be caused by endogenous (e.g. behaviour, contagion, dispersal) or exogenous (environmental gradients) processes [34], [38]. Although conspecific attraction has been described in this species at a local scale [42], we would not expect to find a biological basis for bustard average density in one land mosaic to be influenced by densities in the surrounding mosaics due to behavioural processes, because of the large grain size (thousands of hectares) used in this study. Thus, this spatial dependence was more likely induced by exogenous processes, namely the spatially structured patterns in land cover composition. This is corroborated by the fact that spatial dependence disappeared once the effect of land cover was taken into account in the models.

### Landscape Patterns: Agricultural Intensification does not Decrease Land Cover Diversity

Both intensification and agricultural abandonment in farmed landscapes usually have significant impacts on landscape composition and configuration (e.g., [6], [7]). Their consequence is almost always a trend towards simplification and increased homogeneity, through for example removal of field boundaries and non-crop elements, simplified crop rotations, loss of fallow fields, reduction of crop diversity, or increased field size [6], [43]. This loss of landscape heterogeneity is usually seen as detrimental for biodiversity [43], but there are important exceptions. In Eastern Europe, high biodiversity value grasslands occur as very homogeneous land covers, and increasing agricultural intensification levels will lead to a higher land cover diversity [44]–[45]. This positive correlation between land cover diversity and agricultural intensification was also observed in the current study, where the main gradient of regional variation in landscape composition associated increasing land cover diversity (richness and equitability) with the increased cover by permanent crops and mixed systems. Many of these permanent crops consisted of irrigated olive groves and vineyards more prevalent in an agricultural intensification context. In addition, the obvious gradient of intensification reflected in the second axis of the PCA, expressing the replacement of grasslands by irrigated annual crops, was not related to land cover diversity confirming that, in this geographic context, increased agricultural intensification is not necessarily reflected in a decrease of land cover diversity.

### Little Bustard Densities are Influenced by Both Landscape Diversity and Amount of Grasslands

Our study showed that land cover composition explained ca. 50% of the regional variability in little bustard densities across agricultural land mosaics in southern Portugal. The initial hypothesis that male little bustard density should be higher in landscape mosaics dominated by grasslands, rather than those with higher land cover diversity, was confirmed, with the main driver of population densities being the proportion of grasslands in the land mosaic (and, inversely, the proportion of irrigated crops). Several smaller scale studies have shown the importance of grasslands as the main habitat for displaying males, and where a higher male density can be found (e.g., [9], [13], [14], [17], [18], [29]). In contrast, irrigated crops are usually unsuitable for displaying males [13], [15], [46].

Although grasslands were a key component in the land cover composition for promoting higher bustard densities, other land cover variables were found to influence population density. In land mosaics with higher land cover richness and diversity, which in our geographical context also had a higher cover by permanent crops and mixed systems, male density declined. This shows an avoidance of the species by diverse land mosaics and contrasts with the results of some studies made elsewhere (e.g., [15], [47]). This apparent contradiction is likely explained by the fact that in other studies higher land cover diversity was usually associated to an increased prevalence of grasslands within a patchy landscape, which was not the case in our land mosaics in southern Portugal, and suggests that the context in southern Portugal is similar to the one of Eastern Europe, where increasing agricultural intensification levels lead to a higher land cover diversity harmful for specialist (often endangered) species in these low-intensity agricultural landscapes [44], [45]. The conclusion that little bustards prefer homogeneous grassland landscapes is corroborated by recent study showing that they occurred in higher densities in larger grassland fields in a region in southern Portugal [19]. Finally, densities were also higher in land mosaics with a higher proportion of cereal and ploughed fields, and a lower proportion of unsuitable forest covers. Cereal fields may be suitable for other parts of the yearly cycle [48], [49], or for nesting females [50], thus the existence of some cereal fields in a grassland landscape context might provide additional food and habitat resources to little bustards.

The unexplained regional variability in male density can be due to different unmeasured factors. Habitat quality could play a major role, and it can be expressed as variation in vegetation structure and food availability (e.g., [13], [15], [50]), grazing intensity, human disturbance (e.g., [48]) or a more suitable spatial configuration of the different land cover types. The variable size of our land mosaics may also explain some of this regional variability, if little bustards responded differently at different scales (e.g. 2500 ha c.f. ca. 10000 ha (the size of our largest land mosaic)). This potential scale effect is however unlikely in our dataset, as 80% of the mosaics had a similar size.

### Implications of Land Cover Changes in the Mediterranean for Little Bustard Populations

Land cover changes have strong implications on biodiversity patterns, particularly in the Mediterranean region (e.g., [1], [8]). Feranec et al. [3] described recent land cover changes (1990–2000) in European landscapes and identified the main landscape processes occurring during this period: urbanization, intensification of agriculture, extensification of agriculture, afforestations, deforestation and construction of water bodies. Of these processes, the ones more common in Portugal were afforestations (increase of forest cover due to natural regeneration and plantations), intensification of agriculture (mostly changes of arable land to vineyards, orchards, greenhouses and other irrigated crops.) and deforestation (loss of forest cover by clear-cutting, forest fires, etc.). The results of the current study suggest that the first two processes have caused habitat degradation and loss for little bustards in the last decades, whereas the impact of the latter depends on the type of land cover change that forests are experiencing (if there forests have been replaced by agricultural land, that may have been beneficial for the species). A more detailed study carried out for the period 1985–2000 in Portugal [51] revealed a 4% increase in permanent crops, a 28% decline in the area of pastures and a 2.8% increase in forests. As a whole, landscape fragmentation has increased (more polygons and less area per polygon). Land cover diversity had a large increase, more noticeable in the southern part of the country where this study was undertaken. This was accompanied by a large decline in the land cover dominance index in the region [51]. All these changes also point out to a likely degradation of overall suitability of the landscape mosaic for the little bustard populations, that is likely to continue in the near future and raises concern on the impact of these changes on population size and trends, particularly in Portugal and Spain, which hold more than half of the world’s population of this species [52]. Thus, agri-environmental policies aimed to conserve little bustard populations should aim at maintaining or promoting vast expanses of grasslands in agricultural land mosaics.

## Supporting Information

Table S1 **Land cover level 3 CORINE categories pooled for creating each category used in this study.**
(DOC)

Table S2 **Comparison of different variance structures in the random part of the model.** For each structure, the AIC of the model is given. The fixed part of the model included all explanatory variables.
(DOC)
