# Supplementary material for: Mosaic-Level Inference of the Impact of Land Cover Changes in Agricultural Landscapes on Biodiversity: A Case-Study with a Threatened Grassland Bird
Source: PLoS One. 2012 Jun 18;7(6):e38876. doi: 10.1371/journal.pone.0038876 (PMC3377729; doi:10.1371/journal.pone.0038876)
Supplement: Table S1 — Land cover level 3 CORINE categories pooled for creating each category used in this study. (DOC) [file pone.0038876.s001.doc]

Table S1. Land cover level 3 CORINE categories pooled for creating each category used in this study.

| CORINE categories | Land cover category |
| --- | --- |
| - Non-irrigated arable land [2.1.1] - Pastures [2.3.1] | Dry crops |
| - Permanently irrigated land [2.1.2] - Rice fields [2.1.3] | Irrigated crops |
| - Agro-forestry areas [2.4.4] | Agro-forestry |
| - Vineyards [2.2.1] - Fruit tree plantations [2.2.2] - Olive groves [2.2.3] - Annual crops associated with permanent crops [2.4.1] | Permanent crops |
| - Moors and heathland [3.2.2] - Sclerophyllous vegetation [3.2.3] - Transitional woodland-shrub [3.2.4] - Land principally occupied by agriculture, with significant areas of natural vegetation [2.4.3] | Shrublands |
| - Broad-leaved forest [3.1.1] - Coniferous forest [3.1.2] - Mixed forest [3.1.3] | Forests |
| - Complex cultivation patterns [2.4.2] | Mixed systems |
