# Supplementary material for: Mosaic-Level Inference of the Impact of Land Cover Changes in Agricultural Landscapes on Biodiversity: A Case-Study with a Threatened Grassland Bird
Source: PLoS One. 2012 Jun 18;7(6):e38876. doi: 10.1371/journal.pone.0038876 (PMC3377729; doi:10.1371/journal.pone.0038876)
Supplement: Table S2 — Comparison of different variance structures in the random part of the model. For each structure, the AIC of the model is given. The fixed part of the model included all explanatory variables. (DOC) [file pone.0038876.s002.doc]

Table S2. Comparison of different variance structures in the random part of the model. For each structure, the AIC of the model is given. The fixed part of the model included all explanatory variables. Models are ordered by increasing AIC.

| Variance structure | Description | AIC |
| --- | --- | --- |
| varComb | combination of varIdent + varExp | 324.0 |
| varIdent | Different spread per stratum (year) | 331.1 |
| varExp | exponential of the variance covariate PC 2 | 331.1 |
| none | - | 335.1 |
| varPower | power of the variance covariate PC 2 | 337.0 |
| varConstPower | constant plus power of the variance covariate PC 2 | 339.0 |
| varFixed | fixed variance (proportional to PC 2) | 366.3 |
|  |  |  |
